# Immune responses in aging adults

**DOI:** 10.1172/JCI206227

**Published:** 2026-06-15

**Authors:** Cornelia M. Weyand, Jörg J. Goronzy

**Affiliations:** 1 Department of Medicine and; 2 Department of Cardiovascular Medicine, Mayo Clinic Alix School of Medicine, Rochester, Minnesota, USA.; 3 Department of Immunology, Mayo Clinic College of Medicine and Science, Rochester, Minnesota, USA.; 4 Stanford University School of Medicine, Stanford, California, USA.

## Abstract

As a widely distributed network of cells, tissues, and organs, the human immune system is profoundly vulnerable to the effects of aging. Intrinsic and extrinsic stressors progressively erode its structural integrity and functional resilience, weakening core protective responses and increasing susceptibility to infection, malignancy, and tissue degeneration. At the same time, aging heightens the risk of chronic inflammation and autoimmune disease. Hematopoietic stem cells become uniquely compromised as aging intensifies metabolic and replicative stress. Their continuous high-volume turnover results in diminished self-renewal capacity, skewed lineage output, and dominance of expanded clones. These changes undermine innate immune competence and amplify inflammatory activity. Adaptive immune function declines with age through coordinated cellular and molecular programs. T and B lymphocytes exhibit a decline in naive cells, progressive loss of stemness, shortened lifespan, and constrained clonal diversity. Aging lymphocytes reconfigure transcriptional networks, undergo widespread organelle dysfunction, develop maladaptive stress responses, and redistribute into noncanonical tissue niches. Collectively, these alterations reduce antigen specificity and precision, promote innate-like immune behavior, and confer resistance to tolerance. These mechanisms result in concurrent immunodeficiency and autoimmunity, exemplified by two autoimmune diseases disproportionately affecting older adults: rheumatoid arthritis and giant cell arteritis.

## Introduction

Over the past century, human life expectancy and health span have risen dramatically, marking one of the most profound demographic and biological transformations in modern history. This unprecedented extension of lifespan presents major societal and biomedical challenges and has intensified efforts to understand the molecular and cellular mechanisms that drive aging. Such work has identified a set of conserved “hallmarks of aging”: genomic instability, telomere attrition, epigenetic alterations, loss of proteostasis, impaired autophagy, dysregulated nutrient sensing, mitochondrial dysfunction, cellular senescence, stem cell exhaustion, altered intercellular communication, chronic low-grade inflammation (“inflammaging”), and microbial dysbiosis (Table 1) (1). Together, these processes reflect cumulative genomic damage, disrupted stress responses, defective maintenance of the proteome, impaired bioenergetics, and aberrant communication between cells and tissues.

The immune system — comprising numerous specialized, rapidly adaptable, and migratory cell types — is particularly vulnerable to age-related decline. Immune cells face continuous external and internal stressors and depend on tightly regulated proliferative and metabolic programs. Signs of immune aging appear early, beginning with thymic involution during adolescence (2). Recent proteomic analyses profiling aging across tissues identified a systemic inflection point around age 50, with immune organs such as lymph nodes and spleen among the earliest to exhibit molecular signatures of aging (3, 4). Vascular tissues also showed pronounced age susceptibility, underscoring the interdependence of vascular and immune aging.

Recent epidemiological studies have pinpointed an apparent paradox in immune aging. Most autoimmune disorders have a disease onset of after age 50 years, and incidence rates increase into the seventh and eighth decades of life, a period characterized by measurable declines in immune competence. Of the 19 most prevalent autoimmune conditions, only type 1 diabetes is confined largely to childhood and adolescence (5). The striking observation that aging T cells lose effectiveness in pathogen and tumor immunity yet gain pathogenic potential in autoimmunity has prompted investigation into how aging reshapes T cell function, resilience, and effector programming. Autoimmune diseases have emerged as powerful model systems for defining molecular pathways of immune aging.

**Figure 1:**
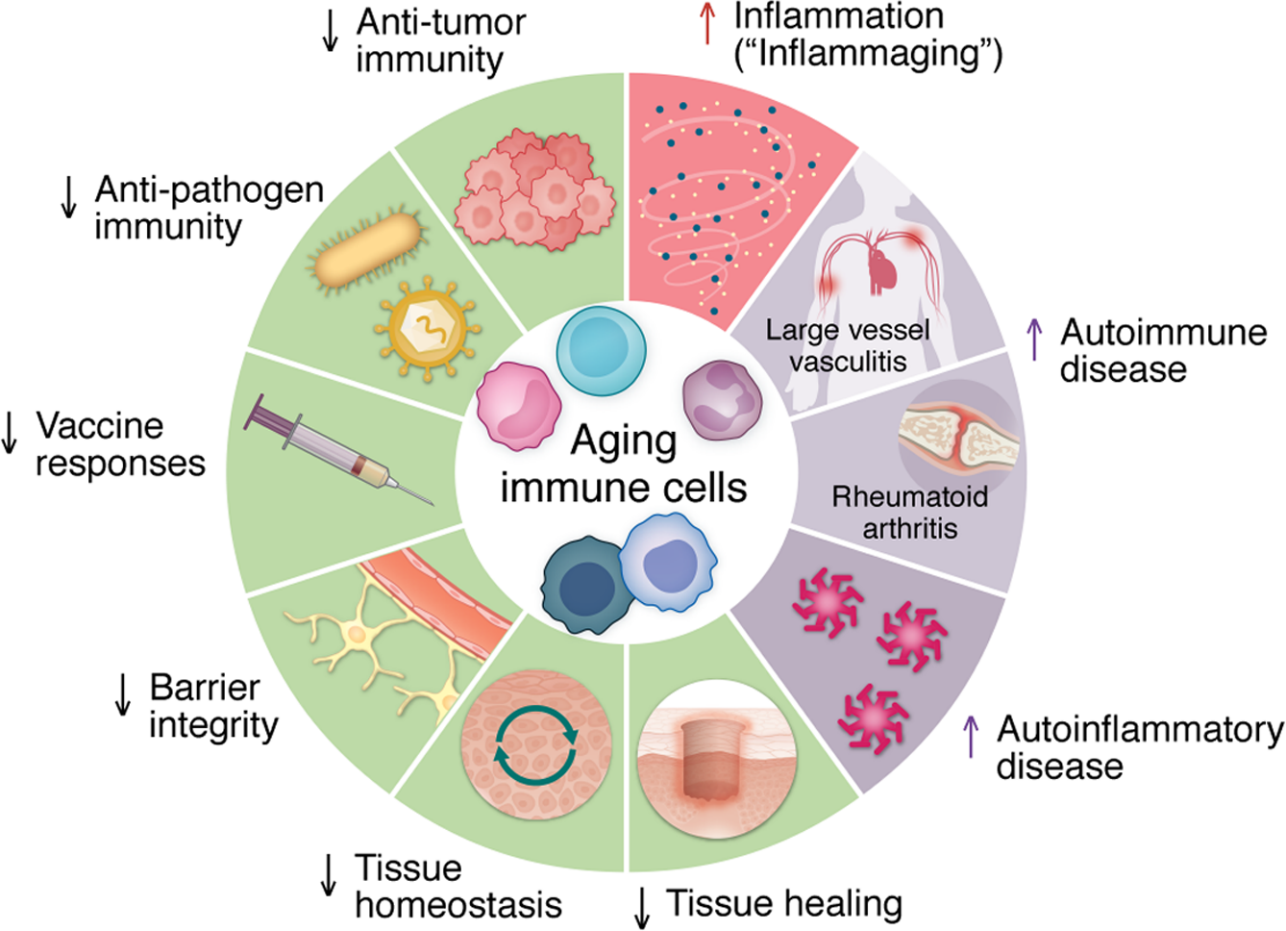
Domains of immune aging. With advancing age, many protective functions of the immune system — such as antitumor surveillance, antimicrobial defense, barrier integrity, and wound healing — progressively decline. Aging affects both innate and adaptive immune cells, making them more prone to generating inflammatory responses. As a result, the aging host faces increased susceptibility not only to infections and malignancies but also to chronic inflammation and a heightened risk of autoimmune disease.

The immune system has diverse functional domains (Figure 1), most of which decline with age: older adults show reduced tumor surveillance, weakened antimicrobial defenses, poor vaccine responses, slower wound healing, and increasingly fragile barriers. In contrast, autoreactivity and chronic low-grade inflammation (“inflammaging”) rise, affecting virtually all organs. The immune system’s broad cellular diversity and complex receptor and signaling networks create multiple points where aging impairs function. In this Review, we highlight recent advances that illuminate how immune aging contributes not only to immune dysregulation and functional decline but also to the onset and progression of autoimmune disease.

## Mechanisms of immune system aging

### Immune cell replication demand.

A central driver of immune cell aging is the continual need to generate new immune cells. A recent study estimated a total immune cell pool of approximately 1.8 × 10^12^ cells in a 73 kg reference adult, with a combined mass of approximately 1.2 kg (6). Lymphocytes and neutrophils each account for approximately 40% of the pool, while monocytes, macrophages, and dendritic cells (DCs) represent smaller fractions. These populations occupy distinct anatomical niches: neutrophils are largely confined to bone marrow reservoirs, whereas T and B lymphocytes predominantly reside in secondary lymphoid organs. Circulating blood remains the most accessible compartment for clinical immunophenotyping of both myeloid and lymphoid lineages.

Despite limited data on how absolute immune cell numbers change with age, current models suggest that lymphocyte pools remain within relatively stable ranges across adulthood, implying constant replenishment (7). Stable-isotope tracing studies demonstrated substantial turnover in young and older individuals (Table 2) (8). Thymic output contributes minimally to maintaining T cell numbers once early adulthood is reached, and homeostatic proliferation of postthymic T cells becomes the dominant mechanism of T cell renewal. Daily production rates of naive T and B cells are each estimated at approximately 65 million to 70 million cells. Memory T cell production is markedly higher, with approximately 600 million CD4^+^ memory T cells and approximately 60 million memory B cells generated per day (8), underscoring the substantial proliferative burden on the CD4^+^ memory compartment.

Replication-driven aging is even more pronounced within the myeloid system. Myeloid cells have much shorter lifespans than lymphocytes, and meeting the enormous demand for neutrophils and monocytes requires extensive proliferation of HSCs. Aging is associated with increasing clonal expansion of HSCs that develop a cell-intrinsic bias toward the myeloid lineage and diminished lymphoid potential (9). Normal HSCs progressively lose fitness due to DNA damage, epigenetic drift, metabolic stress, and niche deterioration, whereas mutated HSCs can gain competitive advantages (10–12). Mutations that enhance self-renewal (*DNMT3A*, *ASXL1*), promote inflammatory resilience (*TET2*), increase proliferation (*JAK2* *V617F*), or bypass senescence (*TP53*) (13) drive clonal expansions, clinically recognized as clonal hematopoiesis of indeterminate potential (10). Low-grade inflammation is a characteristic hallmark of the aging process, with numerous cell types and mechanisms contributing, including end-differentiated immune cells and senescent cells (14). Epigenetic poising of inflammatory genes and organelle dysfunction induce the production of TNF and IL-6 (15, 16), cytokines that are instrumental in age-associated autoimmunity such as in rheumatoid arthritis (RA) and giant cell arteritis (GCA) (17, 18). Inflammatory cytokines further accelerate the process of clonal hematopoiesis by damaging healthy HSCs while preferentially selecting clones resistant to inflammatory stress, for example, *TET2* mutant (enhanced survival in IL-6– and TNF-rich environments) and *DNMT3A* mutant HSCs (stress-induced self-renewal) (19). Thus, inflammaging functions as a potent selective pressure, not solely a mutagenic force.

### Immune system decentralization.

A hallmark of immune aging is the progressive decentralization of immune architecture, in which T and B cells leave their primary tissue and increasingly occupy nonclassical anatomical niches (20, 21) (Table 1). A well-defined example is the formation of tertiary lymphoid structures (TLS), perivascular clusters of T and B cells that arise outside of lymph nodes, spleen, and bone marrow. TLS contain stromal elements resembling those of lymph nodes and may form rudimentary T and B cell zones, yet they never achieve the full organizational complexity of secondary lymphoid organs. With advancing age, TLS can form across virtually all organ systems, most frequently adjacent to large blood vessels in the lung, kidney, and adipose tissues (22, 23). TLS formation is a hallmark of some autoimmune diseases, particularly occurring in the periaortic tissue in GCA (24). They can also be found in the synovium of a subset of patients with RA, usually patients with more severe disease (25–28).

A second manifestation of decentralization is the expansion of tissue-resident memory T cells (TRMs) (29–31). TRMs are a specialized subset of memory T cells that permanently seed tissues such as the skin, lung, gut, liver, and female reproductive tract. Rather than recirculating, they remain embedded within nonlymphoid environments. TRMs maintain a distinct transcriptional program aligned with their defining properties: durable tissue residency, lack of egress, and the capacity to deliver rapid in situ immunity upon antigen reencounter (32).

Decentralization is equally prominent in B cell aging, exemplified by the emergence of aging-associated B cells (ABCs). Unlike classical follicular or marginal zone B cells that reside within germinal centers or B cell follicles, ABCs accumulate in extrafollicular and interfollicular regions, including the splenic marginal zone. ABCs appear progressively with age and expand markedly under conditions of chronic immune stimulation. They are phenotypically defined by CD11c and T-bet expression and reduced CD21, distinguishing them from the classical follicular and marginal zone B cell lineages (33, 34).

ABCs arise in response to persistent antigenic stimulation and to type I and type II IFN- and TLR7/TLR9-driven signals (34). Their transcriptional program reflects diminished dependence on B cell receptor signaling, increased reliance on TLR signaling, and a strong propensity to produce pro-inflammatory cytokines. Functionally, ABCs show impaired germinal center participation and reduced ability to generate high-affinity antibodies. Conversely, they are efficient antigen-presenting cells and potent activators of autoreactive T cells (35).

These features place ABCs at the intersection of immunosenescence and autoimmunity. While they accumulate as part of normal aging, their inflammatory and antigen-presenting activities create conditions conducive to the breakdown of self-tolerance. Consequently, ABCs are increasingly viewed as central contributors to age-related immune dysfunction and to the heightened susceptibility to autoimmune disease in older adults (35, 36).

### Proaging tissue environments.

Because the immune system is broadly distributed and its cells are highly mobile, aging reshapes not only immune cells themselves but also the stromal niches, extracellular matrix, chemokine milieus, and tissue architecture that regulate immune function. These tissue-level alterations are now recognized as major drivers of immunosenescence and inflammaging.

The most classical — and dramatic — example of a proaging immune environment is thymic involution. Age-dependent loss of thymic epithelial cells leads to architectural collapse, profound reductions in naive T cell output, and eventual contraction of the T cell repertoire (37). This process illustrates how decisively tissue aging shapes the quantity and diversity of immune cells available across the lifespan.

Lymph nodes undergo fibrosis and lipomatosis with advancing age, progressively replacing functional stromal tissue with fat (38). This distorts nodal architecture, disrupts migratory pathways, and erodes chemokine gradients required for lymphocyte entry and spatial organization. Consequently, naive T and B cell homing declines, antigen delivery becomes inefficient, antigen presentation is compromised, and germinal center responses deteriorate, weakening both primary and memory immunity (39).

Among stromal populations, fibroblastic reticular cells (FRCs) appear particularly vulnerable to age-related decline (40). FRC networks lose structural integrity and their ability to provide key survival factors — including IL-7 — to maintain the conduit system that transports antigens, and to support the architectural scaffolding required for T cell homeostasis and activation. Lymphatic endothelial cells also exhibit age-related dysfunction, impairing immune cell trafficking into and out of lymph nodes and further compromising immune surveillance (41).

The importance of the tissue environment is equally evident in myeloid aging. The bone marrow niche undergoes structural and metabolic remodeling with age, including loss of osteoblastic support, increased adipogenesis, disordered cytokine networks, and heightened oxidative stress (42). These changes erode the fitness of HSCs while creating a selective advantage for mutated HSCs equipped with enhanced stress response pathways. The aged niche thus becomes a biological bottleneck, favoring expansion of clones able to withstand the hostile environment and promoting the emergence of clonal hematopoiesis.

**Figure 2:**
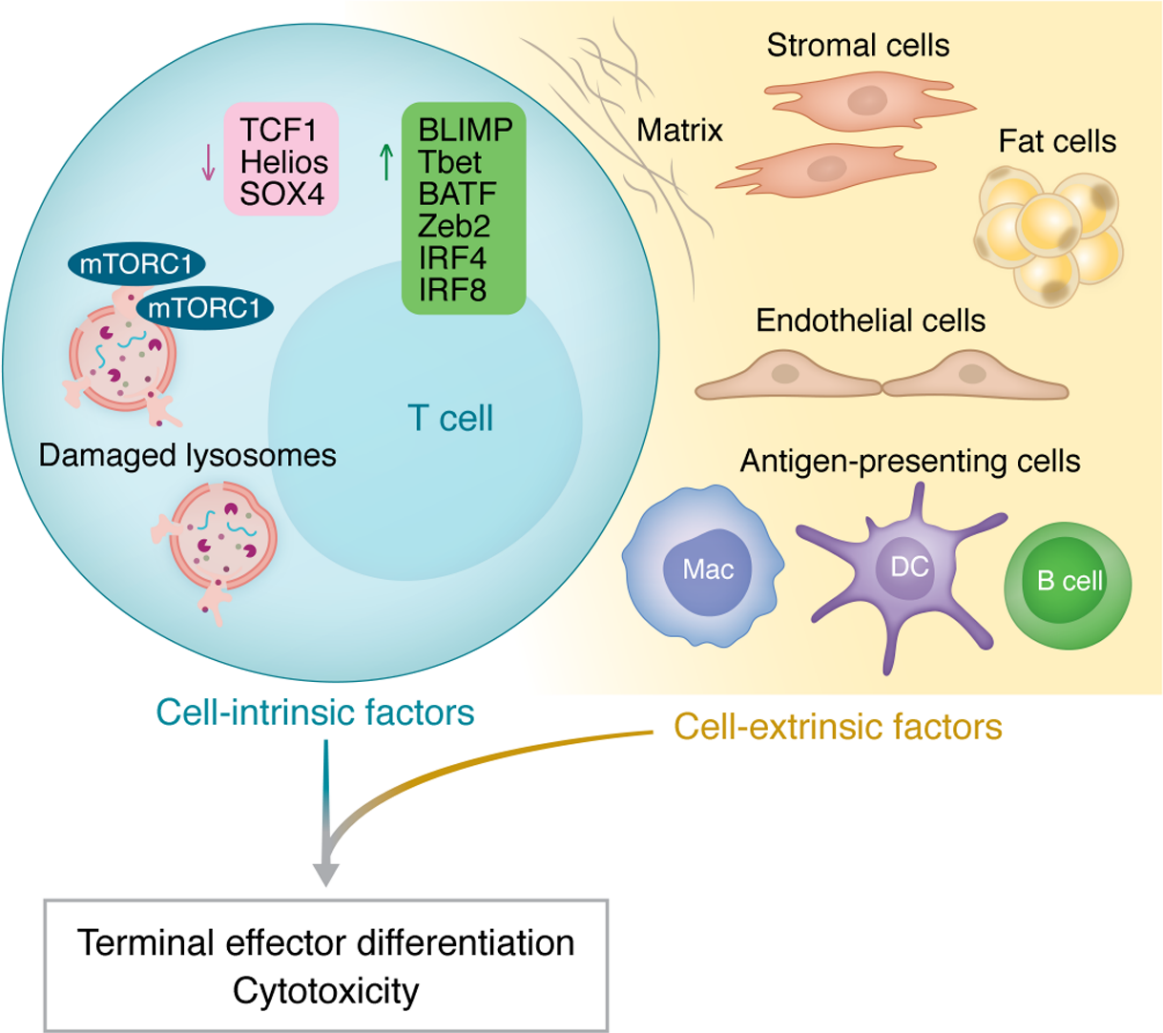
T cell aging: loss of stemness, transcriptional drift, and functional rewiring. T cells possess intrinsic stemness programs and are exceptionally long-lived, yet they are not exempt from aging. Cell intrinsic aging is governed by changes in gene-regulatory networks that preserve T cell stemness and suppress terminal differentiation. With age, key regulators such as TCF1, SOX4, and HELIOS decline. Their loss enables the upregulation of differentiation promoting transcription factors, driving T cells toward irreversible lineage commitment. One downstream consequence is the progressive deterioration of lysosomal function, impairing cellular waste clearance and enabling aberrant mTORC1 activation. This metabolic shift disrupts quiescence and accelerates functional decline. Intrinsic changes are reinforced by extrinsic aging factors arising from tissue and organ aging, including senescent stromal niches and functionally altered antigen presenting cells. Together, these pressures erode tolerance mechanisms and redirect aging T cells toward innate-like and cytotoxic effector phenotypes, reducing immune precision and increasing inflammatory potential.

Growing evidence highlights the profound influence of the metabolic landscapes of tissues — nutrient availability, stromal cell metabolism, lipid composition, and oxidative balance — on immune cell function across organ systems (43) (Figure 2). These factors regulate immune activation, differentiation, and the capacity to maintain homeostasis under stress. Consequently, many age-related, cell-intrinsic defects in myeloid cells, T cells, and B cells are likely compounded, or in some cases initiated, by deterioration of the tissue environments in which these cells reside and function. Age-dependent metabolic changes are therefore emerging as mechanisms guiding the aging immune system to adopt maladaptive and autoimmune responses.

## Aging shapes the T cell landscape in healthy adults

T cells are remarkable among somatic cells because they can persist for years to decades, maintaining protective immunity long after the initial encounter with antigen. A recent study by David Masopust and colleagues, using serial adoptive transfer experiments, provided a striking demonstration of the intrinsic longevity and self-renewal capacity of T cells (44). Memory T cell lineages can survive far beyond the lifespan of the host organism when periodically transferred into a fresh biological environment. This extraordinary durability depends on a coordinated network of metabolic, signaling, epigenetic, and niche-derived support mechanisms. Nevertheless, T cells are not exempt from aging; their aging trajectory results from the combined influence of cell-intrinsic and cell-extrinsic factors, many of which change with age (45, 46).

In the absence of thymic output, naive T cells maintain their numbers through homeostatic proliferation, effectively acting as their own stem cell pool (Table 2) (47). Their aging phenotype is therefore shaped by the efficiency of this proliferative process and by their capacity to preserve stemness. Homeostatic proliferation and survival depend on specialized lymphoid niches in which T cells encounter IL-7 produced by FRCs and receive TCR-mediated tonic signals from self-antigens. Although these niches deteriorate with age (48), naive CD4^+^ T cells exhibit only a moderate decline in cell numbers and TCR repertoire diversity (37). In contrast, naive CD8^+^ T cells decline markedly over time (49, 50). Whether this disparity reflects differences in extrinsic factors — such as the nature of antigen-presenting cells — or intrinsic molecular programs remains unclear. Notably, in mice, the absence of B cells preserves naive CD4^+^ T cell phenotypes and numbers, suggesting that reduced B cell–mediated stimulation protects from aging (51). Likewise, the higher susceptibility of naive CD8^+^ T cells to aging may derive from difficulty maintaining quiescence. Naive CD8^+^ T cells exhibit a lower threshold to activate the Akt/mTORC1 pathway and associated metabolic reprogramming compared with CD4^+^ T cells (50, 52). Aging naive CD8^+^ T cells acquire chromatin accessibility signatures characteristic of differentiation (53). Accordingly, they convert into virtual memory cells under cytokine stimulation, generate granzyme K–expressing populations (age-associated CD8^+^ T cells, Taa cells) that contribute to inflammaging, and accumulate terminally differentiated effector cells expressing granzyme B (54, 55).

Naive CD4^+^ T cells, although more resilient, are not immune to aging (Figure 2). Multiomics studies have revealed extensive age-associated transcriptional remodeling (56). Expression of high-mobility group transcription factors — including TCF1, LEF1, TCF4, SOX4 — as well as HELIOS, all enriched in recent thymic emigrants, declines with age (57, 58). In the context of memory T cell differentiation, these transcription factors maintain stem-like properties that support expansion, self-renewal, and functional plasticity (59, 60). TCF1 plays a central role by suppressing effector differentiation in part by antagonizing the induction of transcription factors such as T-bet, EOMES, BATF, Zeb2, and BLIMP1 that form gene-regulatory networks for various effector functions, including cytotoxicity (61, 62). FOXO1 is a second key regulator of stemness, similarly counteracting effector differentiation and promoting reexpression of TCF1 and IL-7R (63). As a consequence, older naive CD4^+^ T cells preferentially differentiate into effector rather than memory cells (52, 64). Consistent with this interpretation, accumulation of highly differentiated, cytotoxic CD4^+^ T cells is a hallmark of immune aging (65, 66). Their high frequency in supercentenarians indicates a beneficial role (67). Murine studies have shown that loss of cytotoxic CD4^+^ T cells in aged mice worsens senescent cell burden and reduces lifespan (68, 69). However, they are also inflammatory and have been implicated in autoimmune diseases including RA (70–73).

Both TCF1 and FOXO1 are also important for proper organelle function (74, 75). Increased FOXO1 degradation with aging impairs transcription of TFEB and lysosomal enzymes (Figure 2). In parallel, reduced TCF1 expression promotes degradation of the lysosomal proton pump component ATP6V1A. Together, these defects result in impaired lysosomal function, expansion of multivesicular bodies, and increased exocytosis of inflammatory mediators, including mtDNA. Despite these lysosomal defects, mTORC1 activity in naive CD4^+^ T cells from older adults is enhanced though to a lesser degree than in CD8^+^ T cells (52, 76).

Collectively, the age-related reduction of gene-regulatory networks endowing stemness provides a mechanistic explanation for many of the functional deficits observed in T cells from older individuals.

Memory T cells, unlike naive T cells, form a highly dynamic population sustained by cytokine-driven homeostatic proliferation and intermittent bursts of antigen-mediated expansion. Most memory T cells are short-lived, turning over every few months, yet immune memory persists through their continuously renewing progeny, exposing them to selection pressures. This progeny is long-lived: identical twin studies have demonstrated that clonal sister pairs originating in fetal life can persist into old age (77), and murine iterative infection models show that memory lineages remain functional through more than 50 rounds of stimulation (44). However, durability and functionality vary with experimental conditions, and accordingly, a striking feature of memory T cell biology is the heterogeneity of aging trajectories across antigen-specific lineages. Varicella zoster virus–specific T cells exhibit a progressive loss of stem-like features and a shift toward innate-like transcriptional states in older adults (78). Vaccination does not reverse these defects, but rather induces a Th17 subset with superior functional signatures. CD8^+^ T cells specific to different EBV epitopes show similarly varied patterns: some lineages develop gradually over decades into TEMRA cells, others transition early to this state, and some appear resistant to aging (79). Likewise, influenza-specific CD8^+^ T cells do not undergo the terminal differentiation seen in the global CD8^+^ population (80). TEMRA cells exhibit innate-like transcriptional signatures overlapping with NK cell profiles (81). Although they bear selected features of senescence, they are not fully senescent or exhausted (79, 82). They arise predominantly among CD8^+^ T cells under chronic latent viral stimulation, but aging CD4^+^ T cells also develop innate-like qualities, including perforin and granzyme B expression (65, 67). Consistent with their cytotoxic abilities, these end-differentiated cells have transcription factor profiles including T-bet, Zeb2, BLIMP1, and EOMES, which identifies them as Th1 cells (Figure 2). However, multiomics studies have also identified enrichment of a CD4^+^ T cell population with age that has transcriptional hallmarks of Th2 cells (56, 83). TEMRA cells differ from CD8^+^ Taa cells, which are marked by granzyme K expression (84). Although Taa cells share aspects of exhaustion, they are not clearly linked to chronic infection and appear to contribute to inflammaging.

Together, these findings illustrate that CD4^+^ T cells are much more resilient to aging than CD8^+^ T cells. While naive CD8^+^ T cells are largely lost with age, naive CD4^+^ T cells are maintained with age and continue to be diverse. While they are still functional, they have rewired their transcription factor network to be poised to differentiate into effector cells. Memory T cell aging is highly context dependent, shaped by antigen specificity, exposure history, and lineage-intrinsic transcriptional programs. This heterogeneity helps explain why aging does not uniformly degrade memory immunity and why certain antigen-specific T cell pools remain functionally robust, whereas others transition toward innate-like, inflammatory, or senescent-skewed states. Such diversity in aging trajectories has important implications for immune resilience in older adults and for understanding how age-associated inflammatory diseases emerge from dysregulated memory T cell populations.

## RA as a model of aging-associated autoimmunity

Among the 22 million individuals enrolled in the UK Biobank, the median age at RA diagnosis is 65 years (5, 85), with approximately 75% of cases diagnosed between ages 50 and 71. This age distribution underscores a central biological insight: RA typically becomes clinically overt during the second half of life, a period marked by measurable decline in adaptive immune competence. The dominant genetic risk for RA resides in the HLA class II region, which encodes molecules engaged in presenting antigenic peptides to CD4^+^ T cells (86, 87). Beyond HLA, the major RA genetic risk polymorphisms, including variants in *PTPN22*, *CTLA4*, *STAT4*, *CD40*, *TRAF1*, *C5*, and *PADI4* (88), all converge on pathways integral to T cell biology (89, 90), such as T cell activation, costimulation, and intracellular signaling. Consequently, RA offers a uniquely powerful model for investigating how immune aging, particularly aging of the T cell compartment, predisposes individuals to autoimmunity.

**Figure 3:**
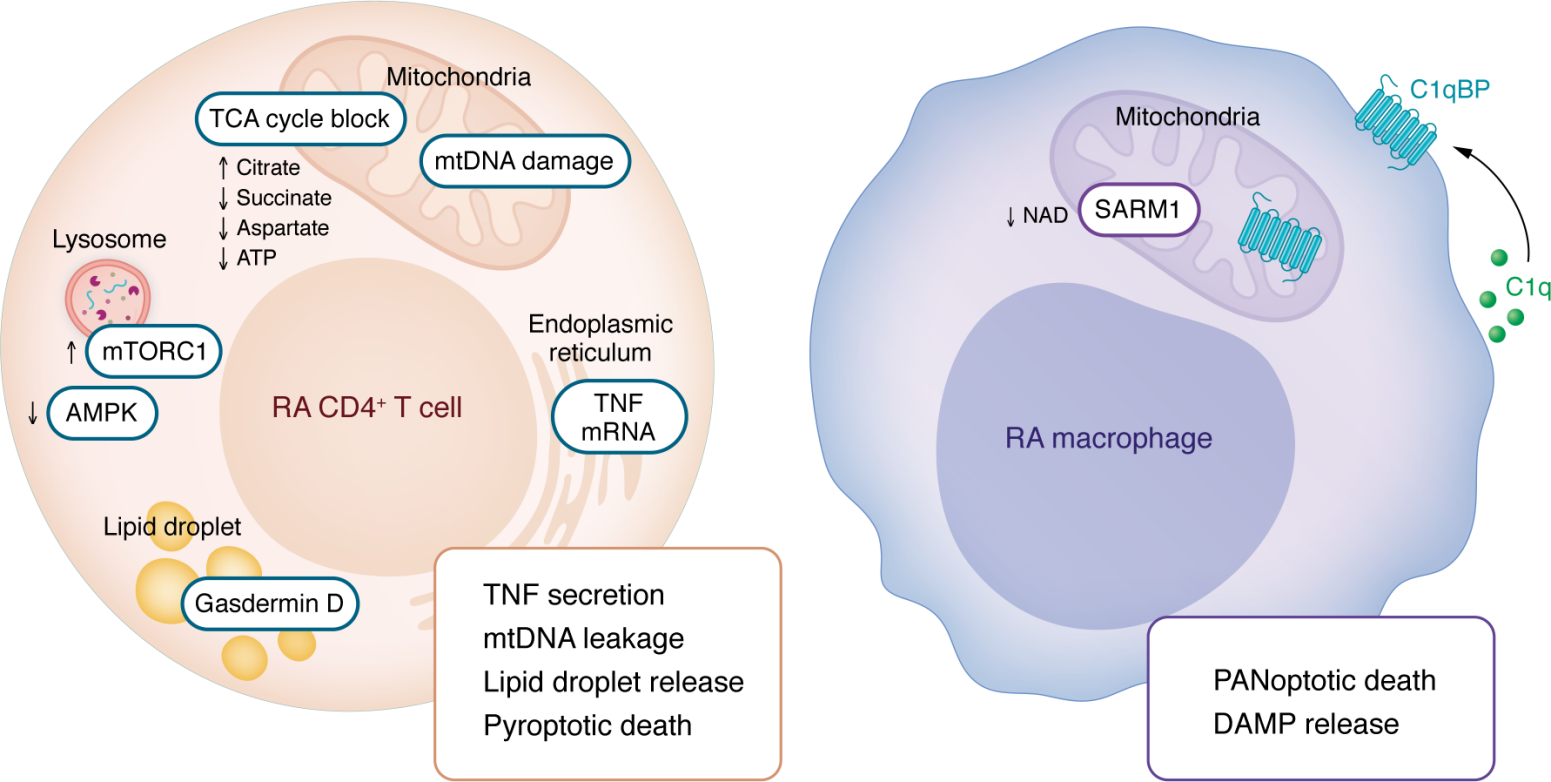
Signatures of T cell and macrophage aging in RA: organelle stress, metabolic exhaustion, and pro‑inflammatory cell death. Aging of the immune system predisposes the host not only to impaired protective responses but also to the development of autoimmune disease. RA exemplifies this paradox. CD4^+^ T cells and synovial macrophages from patients with RA exhibit multiple hallmarks of immune aging, including declining organelle function and loss of coordinated interorganelle communication. In CD4^+^ T cells, defective mtDNA repair undermines ATP generation and disrupts TCA cycle activity. Compromised mitochondrial fitness places secondary stress on the ER and lysosomes, promoting the accumulation of lipid droplets. In RA, these lipid droplets serve as “killer organelles” by packaging the pore‑forming molecule gasdermin D. Synovial macrophages undergo a distinct form of mitochondrial collapse driven by C1q‑dependent activation of the NADase SARM1. This pathway triggers inflammasome activation and culminates in PANoptotic cell death. Release of cytosolic and nuclear material from dying CD4^+^ T cells and MerTK^+^ macrophages amplifies synovial tissue inflammation and perpetuates disease.

Early molecular analysis of CD4^+^ T cells from patients with RA revealed a phenotype of accelerated immunological aging: premature loss of CD28 expression, age-inappropriate telomere erosion, and a contraction of clonal diversity (70, 91–94). Together, these features indicate that RA T cells experience the liabilities of aging well before chronological aging would predict. Subsequent work has dissected the underlying mechanisms. Aged T cells acquire pathogenic effector functions not simply because they accumulate damage but because aging fundamentally reshapes how intracellular organelles communicate, coordinate metabolism, and maintain homeostasis. In this model, organelle stress involving mitochondria, lysosomes, lipid droplets and the ER, and the breakdown of organelle crosstalk reprogram T cells toward pro-inflammatory, tissue-destructive states (95, 96). Rather than remaining quiescent, these T cells are pushed into maladaptive activation loops that initiate and perpetuate chronic inflammation. This conceptual framework brings RA pathogenesis into close alignment with the hallmarks of aging: genomic instability affecting nuclear DNA, telomeric DNA, and mtDNA; impaired proteostasis driven by defective autophagy and lysosomal dysfunction; dysregulated nutrient sensing and metabolic inflexibility; and disturbances in cell-cell communication that impair tissue tolerance (Figure 3).

### Sick mitochondria drive inflammation through lytic cell death.

In RA, CD4^+^ T cells show profoundly impaired mitochondrial health rooted in defective mtDNA repair (97–100) (Figure 3). Although the nucleus produces the necessary repair machinery, including the nuclease MRE11A, RA-derived CD4^+^ T cells fail to import these molecules into mitochondria, leaving mitochondrial genomes vulnerable (97). Insufficient mtDNA repair disrupts two major domains of cellular homeostasis (101, 102). First, defective mtDNA maintenance undermines metabolic competence by impairing electron transport chain integrity and TCA cycle activity, limiting respiration and bioenergetic flexibility. Second, fragments of mtDNA escape into the cytosol and extracellular space and act as potent damage-associated molecular patterns (DAMPs) that trigger local inflammation and amplify immune activation (97). Thus, mtDNA repair is essential for maintaining T cell metabolic fitness and preventing inflammation driven by mitochondrial nucleic acid release.

Given inadequate mtDNA repair in RA CD4^+^ T cells, mitochondrial ATP production falls sharply (103), and TCA cycle activity becomes fundamentally disrupted (17, 104, 105). RA CD4^+^ T cells contain unusually low levels of late-cycle metabolites such as succinate, malate, and oxaloacetate, and they generate insufficient aspartate (17), an amino acid needed for metabolic signaling and ER coordination. In contrast, early TCA intermediates acetyl-CoA, citrate, and α-ketoglutarate build up, consistent with a partially reversed TCA cycle reoriented toward lipogenesis (106, 107). Excess acetyl-CoA fuels posttranslational modifications, including protein acetylation (108). Hyperacetylation of cytoskeletal proteins disrupts cytoskeletal structure, mispositioning organelles and altering cell morphology. Together, these metabolic and structural defects render RA CD4^+^ T cells highly mobile and tissue invasive (106).

Chronic pressure on mitochondrial energy output, loss of mitochondrial adaptability, and resulting metabolic crisis are also evident in synovial macrophages from patients with RA (109–111). These macrophages coordinate increased antigen-presenting capacity and associated bioenergetic needs through induction of the transcription factor RFX5, which selectively upregulates glutamate dehydrogenase 1. The enhanced glutamate utilization simultaneously intensifies mitochondrial stress. Aggressive RA synovitis contains a heterogeneous population of macrophages (112, 113), including SelenoP^+^CD206^+^MerTK^+^ macrophages (110), a population traditionally viewed as tissue protective. However, in RA, these macrophages are prone to lytic cell death and release of intracellular contents. Recent mechanistic work explains this paradox. Secretome analyses identified C1q as a dominant product of SelenoP^+^CD206^+^MerTK^+^ macrophages. These cells both secrete C1q and sense it through the receptor C1QBP. Persistent autocrine C1q exposure drives C1q–C1QBP complexes into mitochondria, where they engage the matrix enzyme SARM1, triggering NAD^+^ degradation and a surge in cyclic ADP-ribose (cADPR) production (110). Elevated cADPR promotes inflammatory caspase activation and PANoptosome assembly. Through this pathway, C1q sensing initiates a lytic, pro-inflammatory death program, amplifying synovial inflammation. Ultimately, tissue-resident macrophages under chronic stimulation undergo programmed self-destruction, releasing DAMPs (Figure 3).

Together, loss of metabolic resilience is a shared vulnerability across adaptive and innate immune cells in RA. Metabolic exhaustion precedes nonapoptotic, pro-inflammatory cell death (114), illustrating how chronic activation erodes the cell’s capacity to regulate its fate. Rather than preserving metabolic flexibility, CD4^+^ T cells and macrophages exhibit diminished resilience, a hallmark of cellular aging (Figure 3).

### Lysosomal insufficiency promotes pathological mTORC1 activation.

Lysosomes are energy-intensive organelles whose acidity, membrane stability, and degradative functions depend on continuous metabolic support from mitochondria (115, 116). When bioenergetic supply becomes limited, lysosomes lose the ability to sustain pH gradients and membrane integrity, leading to lysosomal dysfunction. Lysosomes act as hubs that integrate metabolic information to directly shape cell fate decisions. This regulatory capacity hinges on the coordinated recruitment of AMPK and mTORC1 to the lysosomal surface (117), where the two kinases mutually modulate each other. Under nutrient-rich conditions, amino acid accumulation within lysosomes activates Rag GTPases, which stimulate mTORC1 to initiate anabolic growth and protein synthesis programs. Conversely, nutrient scarcity triggers AMPK-mediated inhibition of mTORC1, shifting the cell toward energy conservation, mitochondrial activation, and biogenesis (118, 119).

In RA CD4^+^ T cells, communication between mitochondria and lysosomes is disturbed (120, 121). AMPK fails to localize to lysosomes, leaving mTORC1 inadequately restrained (122) (Figure 3). As a result, mTORC1 becomes uncoupled from ATP status, allowing metabolically depleted CD4^+^ T cells to grow and proliferate despite profound bioenergetic stress. Disabling the energy-sensing brake renders these cells vulnerable to metabolic collapse and unable to execute ATP-dependent apoptotic pathways. This paradoxical mTORC1 hyperactivation in energy-poor cells is a hallmark of aged T cells and contributes to their escape from mechanisms that normally limit immune responses.

### Mitochondrial impairment alters ER size and function.

In healthy cells, the ER performs essential functions in protein folding and quality control, calcium storage and release, lipid synthesis, and membrane production. Perturbations in protein-folding load, redox balance, and calcium homeostasis impose ER stress, activating the unfolded protein response (UPR). Through its three sensors — IRE1α, PERK, and ATF6 — the ER monitors cellular metabolic status, including mitochondrial performance, and adjusts proteostasis to maintain survival (123, 124).

ER–mitochondria interfaces, or mitochondria–ER contact sites (MERCs), coordinate rapid exchange of metabolites, lipids, and calcium to integrate mitochondrial bioenergetics with ER homeostasis (125, 126). In RA CD4^+^ T cells, mitochondrial dysfunction provokes an ER remodeling response marked by expansion of ER membranes and reorganization of MERCs (17). Single-cell analyses show an inverse correlation between mitochondrial membrane potential and ER size, particularly the rough ER (17).

Mechanistic studies reveal that specific mitochondrial metabolites — malate, oxaloacetate, and aspartate — regulate ER size. Because RA CD4^+^ T cells operate a reversed TCA cycle, they produce reduced levels of malate and oxaloacetate, disrupting the malate–aspartate shuttle, depleting cytoplasmic aspartate and impairing NAD^+^ regeneration (17).

NAD^+^ deficiency is sensed by the ER through ADP-ribosylation of BiP (17), the central ER chaperone controlling UPR activation. Under stress, BiP disengages from IRE1α, PERK, and ATF6 to bind misfolded proteins, initiating UPR signaling. In RA CD4^+^ T cells, BiP-driven stress responses expand the rough ER, which becomes enriched in ribosomes actively translating TNF mRNA (17) (Figure 3). This leads to persistent TNF overproduction. Through this cascade, from impaired mitochondrial metabolism to ER stress and translational reprogramming, RA CD4^+^ T cells become pathological TNF “superproducers,” perpetuating chronic inflammation.

### Stress responses repurpose lipid droplets into cytotoxic organelles.

ER stress, arising from mitochondrial dysfunction or nutrient deprivation, drives upregulated lipid droplet (LD) biogenesis, with LDs forming directly from the ER membrane (127, 128). In RA CD4^+^ T cells, LD formation serves as an initial adaptive mechanism to limit lipotoxicity, buffer excess free fatty acids (FFAs), and maintain membrane homeostasis. Notably, patient-derived CD4^+^ T cells are primed for LD production, reflected by induction of lipid-handling genes, such as the structural LD protein perilipin 2 (106, 129). However, metabolic profiling of RA CD4^+^ T cells reveals that LDs can shift from protective stores to cytotoxic organelles (130). This transition is driven by the extreme metabolic conditions within inflamed rheumatoid joints. Synovial tissue from patients with RA shows progressive accumulation of FFAs with increasing inflammatory severity (130). CD4^+^ T cells entering this lipid-rich environment cannot accommodate the overwhelming lipid load and instead undergo lipid-induced pyroptotic cell death, marked by gasdermin D–mediated pore formation. Proteomic analyses of LDs from RA CD4^+^ T cells identified gasdermin D and its activating palmitoyltransferase zDHHC5 as LD-associated proteins (130), indicating that LDs serve as reservoirs for the pyroptotic machinery (Figure 3).

Impaired mitochondrial resilience thus initiates a broader maladaptive remodeling program involving ER membrane expansion, accelerated LD generation, gasdermin D loading, and trafficking of gasdermin-rich LDs toward the plasma membrane. This culminates in membrane disruption and release of T cell–derived DAMPs, illustrating how stress-adapted metabolic programs in aged CD4^+^ T cells directly promote inflammatory effector behavior.

Together, these aging-driven defects redirect T cell biology toward pathogenic outcomes. CD4^+^ T cells become hyperresponsive yet functionally destabilized: they misinterpret metabolic cues, overproduce inflammatory mediators, acquire cytotoxic properties, and lose mechanisms that normally suppress autoreactivity. Within inflamed tissue, this creates an immunological ecosystem primed for persistent autoimmunity: one in which metabolically stressed, maladapted T cells act as central drivers and sustainers of chronic inflammatory disease (95, 96, 130–132) (Figure 3).

## The immune aging paradox of GCA

The enrichment of autoimmune disease in older adults has been attributed to age-related immune decline, which weakens the multilayered mechanisms that normally maintain self-tolerance. It is reasonable to assume that tolerance pathways are particularly vulnerable to age-associated deterioration (133, 134). However, emerging evidence reveals a more complex paradigm: incomplete or stalled immune aging can itself promote autoimmunity. In this model, select components of the immune system retain youthful vigor, while other organ systems age and accumulate neoantigens to which the host was never tolerized. This mismatch between immune aging and tissue aging (135) creates fertile ground for autoimmune responses.

Proteomic mapping of human tissues shows that the vascular system, especially the aorta, is highly susceptible to age-related structural and molecular changes (3). Aging aortas exhibit degradation of elastic laminae, progressive fibrosis, and deposition of amyloid species and complement proteins (3). Although autoimmunity targeting the aorta is rare, it emerges in GCA, a vasculitis almost exclusively affecting individuals over 50, with a median diagnostic age near 75 (136). The strict age dependence of GCA underscores the interplay between vascular aging and immune dysregulation (137).

**Figure 4:**
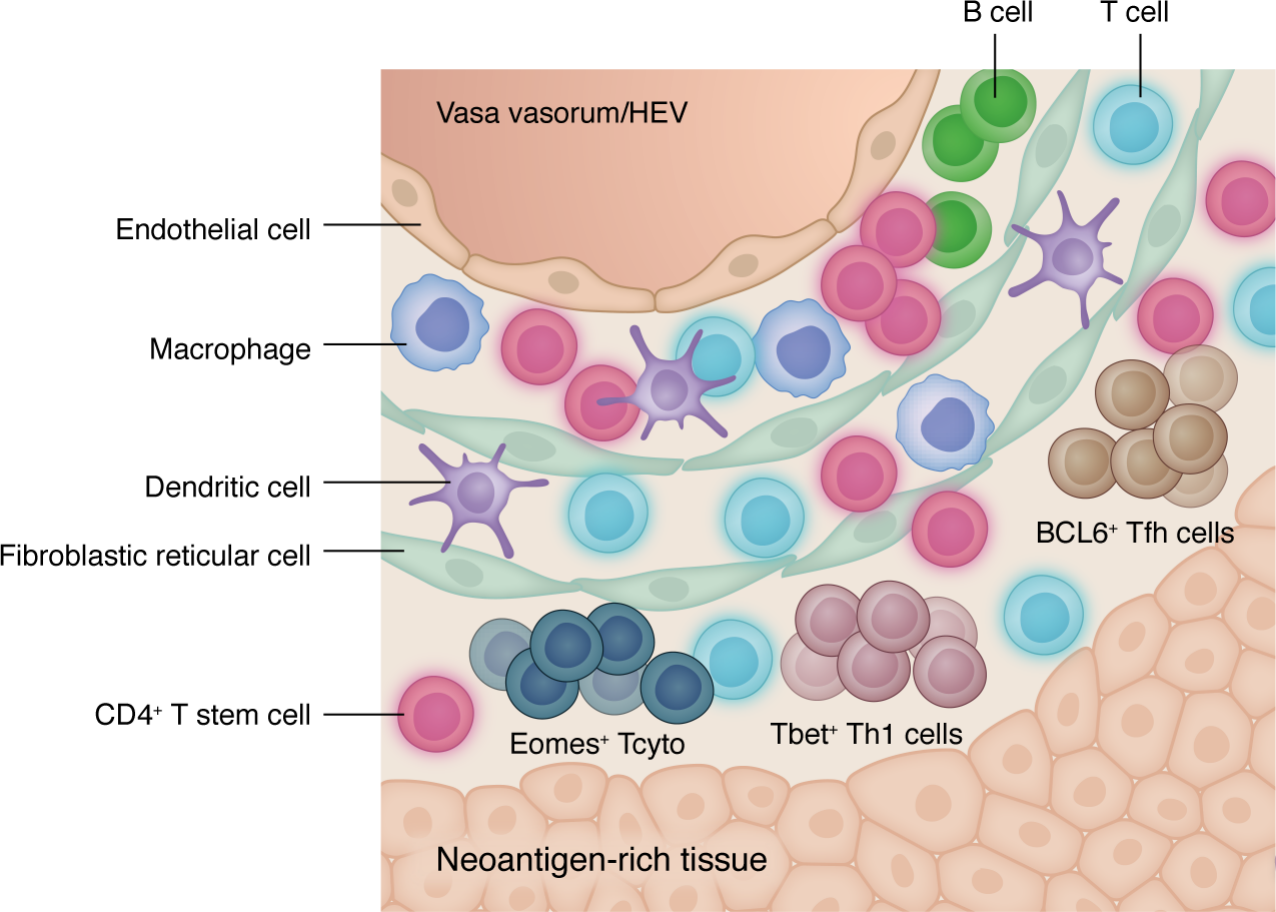
Aging-resistant T stem cells drive autoimmune disease. A subset of individuals is protected from age‑related decline in T cell stemness. In these individuals, aging‑resistant CD4^+^ T stem cells can reside within TLS, where they function as progenitors for differentiated effector T cells that infiltrate neoantigen-rich tissues and precipitate autoimmune pathology. This mechanism of stalled immune aging is exemplified in patients with the autoimmune vasculitis GCA. Persistent T stemness is maintained by a specialized niche located in the perivascular space, formed through interactions of CD4^+^ T stem cells with high endothelial venules (HEVs), fibroblastic reticular cells, and CD11c^+^ dendritic cells. B cells may contribute to these stem cell–supporting lymphoid aggregates but are not essential components of the niche.

Studies in GCA have transformed our understanding of immune aging. Rather than demonstrating uniform immunosenescence, patients with GCA retain a population of stem-like CD4^+^ T cells that preserve proliferative capacity and responsiveness uncommon to advanced age (24) (Figure 4). This preserved T cell stemness enables recognition of neoantigens arising within aging arterial tissue, fueling granulomatous inflammation. These observations suggest a provocative hypothesis: synchronous aging of immune and peripheral tissues may be necessary to maintain tolerance. When immune aging lags behind organ aging, vigorous T cells may interpret aging tissues as foreign, initiating autoimmune attack.

Stem-like CD4^+^ T cells in GCA were identified through analyses of aortic tissue obtained during surgical repair of aneurysms, a complication that reflects chronic aortic inflammation. Surprisingly, histopathology revealed persistent aortitis in most patients, even a decade after diagnosis and presumed remission (138, 139). Immune infiltrates formed complex T cell–rich structures around the vasa vasorum; in some patients, these evolved into TLS, demonstrating extensive lymphoid decentralization within the vessel wall (140).

Single-cell RNA-seq studies identified five CD4^+^ T cell subsets in GCA aneurysms (24), including an unexpected population lacking effector signatures but expressing TCF1, a transcription factor essential for thymic T cell development, maintenance of naive and stem-like memory T cells (141), and long-term self-renewal. Functional assays confirmed that these TCF1^+^CD4^+^ T cells are indispensable for vasculitis in serial transplantation models, fulfilling criteria for immune stem cells. These stem-like T cells depend on highly specialized lymphoid niches for survival. They interact closely with high endothelial venule endothelial cells, follicular reticular cells, and CD11c^+^ DCs (24) (Figure 4). Notably, patients with GCA show aberrant Notch pathway activation, with CD4^+^ T cells expressing excess Notch1 (142) and vasa vasorum endothelial cells expressing its ligand Jagged1 (143), highlighting a key endothelial–T cell communication axis required for sustaining T cell stemness.

Stalled immune aging in GCA is not confined to T cells. Myeloid cells from patients exhibit unusually low expression of the checkpoint ligands programmed cell death ligand 1 (144) and CD155 (145), a pattern more typical of young immune systems. The preservation of youth-like inhibitory profiles in antigen-presenting cells further supports a systemic uncoupling of immune aging from overall organismal aging (135). Why immune aging is selectively delayed in GCA remains unclear, but Scandinavian cohort studies suggest a pre-GCA metabolic phenotype characterized by low body mass index, low blood glucose, and low triglycerides (146), features consistent with an antiaging metabolic profile.

Together, these findings redefine the age-associated autoimmune disease GCA as a disorder driven not simply by immunosenescence but by a discordant aging process: the body ages, and tissues accumulate neoantigens, yet pockets of the immune system retain youthful capacity for activation and clonal expansion. This imbalance fuels pathogenic immunity, illustrating how the retention of stem-like T cells late in life becomes a vulnerability factor for autoimmune disease.

## Conclusions and outlook

Aging progressively erodes immune integrity through stem cell exhaustion, metabolic stress, organelle dysfunction, and declining lymphocyte stemness. Hematopoietic and lymphoid tissues lose regenerative capacity, while maladaptive stress responses imprint both the innate and adaptive immune systems, often driving inflammatory and lytic cell death pathways. As tolerance mechanisms weaken, T and B cells adopt more innate-like features, increasing susceptibility to autoimmunity. Concurrent aging of lymph nodes, bone marrow, and the vasculature further disrupts immune coordination, yielding a system that is simultaneously immunodeficient and hyperinflammatory. Together, these vulnerabilities create the conditions for inflammaging and later life autoimmune disease.

Future therapeutic strategies should aim to restore metabolic resilience, improve organelle communication, and preserve lymphocyte stemness. Interventions that enhance mitochondrial repair, temper persistent mTOR signaling, and correct maladaptive ER stress may help rebalance aging immune cells. A unique opportunity lies in elucidating mechanisms of delayed immune aging, which could guide approaches that rejuvenate immune function, strengthen vaccine responsiveness, and prevent age-associated autoimmunity.

## Conflict of interest

CMW’s laboratory has received support from a research contract with Electra Therapeutics.

## Funding support

This work is the result of NIH funding, in whole or in part, and is subject to the NIH Public Access Policy. Through acceptance of this federal funding, the NIH has been given a right to make the work publicly available in PubMed Central.

- NIH (R01AR042527, R01AI108906, R01HL142068, U01AI179609, R01HL117913 to CMW and R01AI108891, R01AG045779, R01AI184360 to JJG).

## Figures and Tables

**Table 1:**
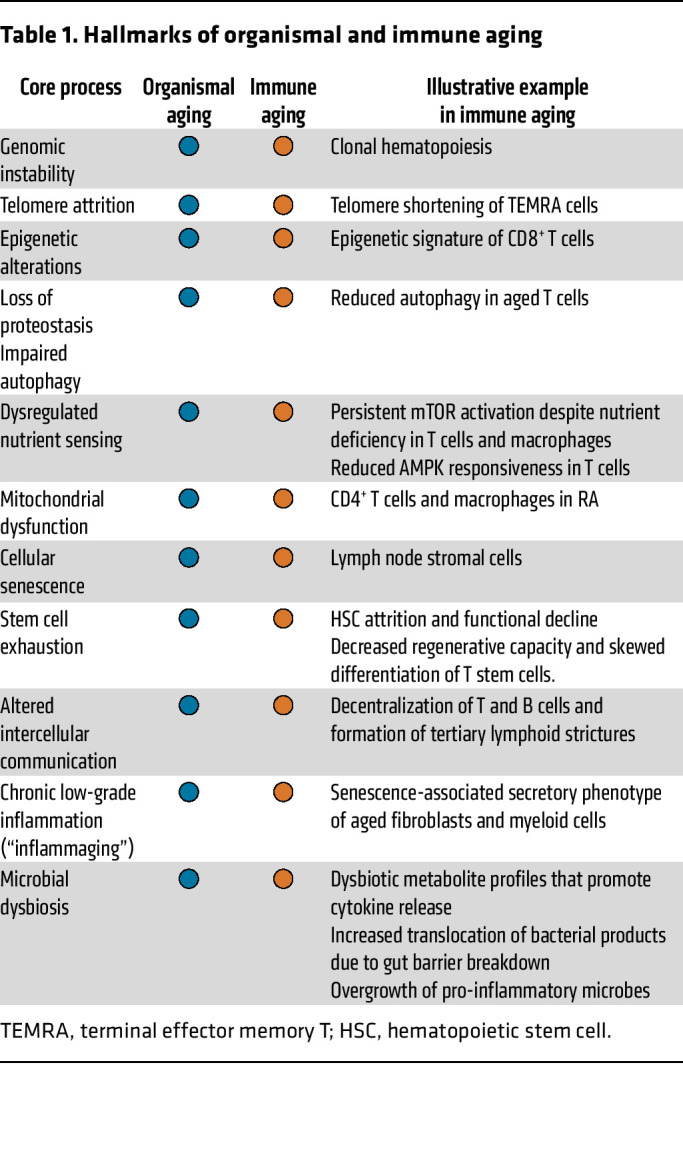
Hallmarks of organismal and immune aging

**Table 2:**
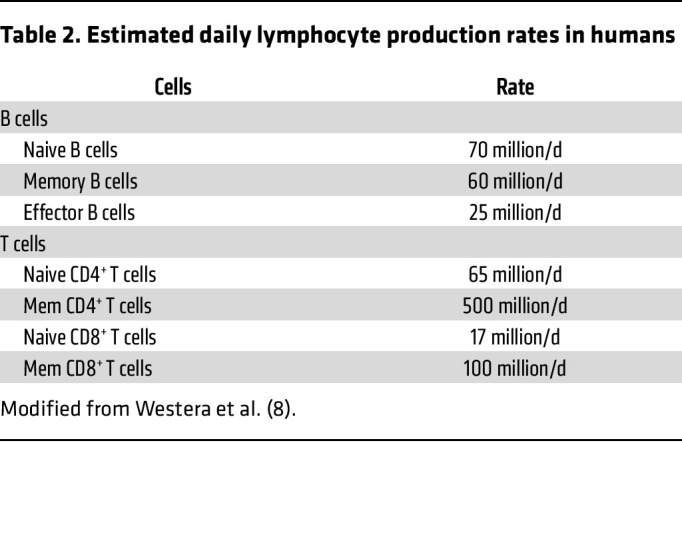
Estimated daily lymphocyte production rates in humans

## Version 1 06/15/2026

Electronic publication
